# Association of isoproterenol infusion during catheter ablation of atrial fibrillation with outcomes: insights from the UC San Diego AF Ablation Registry

**DOI:** 10.1007/s10840-022-01448-x

**Published:** 2022-12-12

**Authors:** Omar M. Aldaas, Douglas Darden, Praneet S. Mylavarapu, Frederick T. Han, Kurt S. Hoffmayer, David Krummen, Gordon Ho, Farshad Raissi, Gregory K. Feld, Jonathan C. Hsu

**Affiliations:** Cardiac Electrophysiology Section, Division of Cardiology, Department of Medicine, University of California San Diego Health System, 9452 Medical Center Drive, 3rd Floor, Room 3E-417, La Jolla, CA 92037 USA

**Keywords:** Catheter ablation, Atrial fibrillation, Isoproterenol, Outcomes

## Abstract

**Background:**

High-dose isoproterenol infusion is a useful provocative maneuver to elicit triggers of atrial fibrillation (AF) during ablation. We evaluated whether the use of isoproterenol infusion to elicit triggers of AF after ablation is associated with differential outcomes.

**Methods:**

We performed a retrospective study of all patients who underwent de novo radiofrequency catheter ablation of AF enrolled in the University of California, San Diego AF Ablation Registry. The primary outcome was freedom from atrial arrhythmias on or off antiarrhythmic drugs (AAD).

**Results:**

Of 314 patients undergoing AF ablation, 235 (74.8%) received isoproterenol while 79 (25.2%) did not. Among those who received isoproterenol, 11 (4.7%) had additional triggers identified. There were no statistically significant differences in procedure time (*p* = 0.432), antiarrhythmic drug use (*p* = 0.289), procedural complications (*p* = 0.279), recurrences of atrial arrhythmias on or off AAD [adjusted hazard ratio (AHR) 0.92 (95% CI 0.58–1.46); *p* = 0.714], all-cause hospitalizations [AHR 1.00 (95% CI 0.60–1.67); *p* = 0.986], or all-cause mortality [AHR 0.14 (95% CI 0.01–3.52); *p* = 0.229] between groups.

**Conclusions:**

In this registry analysis, use of isoproterenol is safe but was not associated with a reduction in recurrence of atrial arrhythmias.

## Introduction

Atrial fibrillation (AF) becomes increasingly prevalent and its burden is expected to continue to grow [1]. Given the potential adverse effects of pharmacologic antiarrhythmic therapy and its inconsistent success at maintaining sinus rhythm, catheter ablation has emerged as a viable alternative for rhythm control of AF. Catheter ablation will likely become even more common due to its inclusion in guidelines and more data emerging in favor of an early rhythm control strategy [2, 3]. Isoproterenol is a cardiac β_1_ and β_2_ adrenoreceptor agonist with positive chronotropic, dromotropic, and inotropic effects [4]. It has been used to assess for non-pulmonary vein triggers of AF after pulmonary vein isolation ablation as it has been shown to be over 80% successful in provoking pulmonary vein and extra pulmonary vein triggers [5]. However, data is lacking on whether the use of ablating triggers identified by isoproterenol is associated with differential outcomes in follow-up after ablation of AF. This is important as isoproterenol can result in hypotension during the procedure and is expensive, with its price increasing over recent years [6].

## Methods

### Study design and registry population

This study was an observational, retrospective cohort study using data collected as part of the University of California (UC) San Diego AF Ablation Registry and approved by the UC San Diego Institutional Review Board. The UC San Diego AF Ablation Registry was designed as a clinical registry of all patients undergoing left atrial ablation procedures for atrial arrhythmias at UC San Diego a single academic center, as captured by a procedural database (Perminova, Inc, San Diego, CA) to collect patient, provider, and intra-procedural characteristics. All AF ablation procedures captured by the registry from October 2009 to March 2015 were linked to clinical encounters as recorded by the electronic medical record at UC San Diego Medical Center (Epic, Verona, WI). Patients with a prior AF ablation procedure were excluded (*n* = 296). Data on baseline demographics, medical history, laboratory data, medications, and cardiovascular implantable devices were collected as part of the UC San Diego AF Ablation Registry. Intra-procedural registry reports were reviewed to determine fluoroscopy and procedure times and ablation lesion sets.

### Patient groups and outcomes

Patients were stratified into groups based on whether they received or did not receive isoproterenol infusion during de novo catheter ablation of AF. Clinical outcomes were determined during all follow-up and included in-hospital adverse events, recurrence of atrial arrhythmia at final follow-up on or off antiarrhythmic drugs (AAD) and off AAD, and all-cause hospitalizations and mortality. The choice to continue or discontinue AADs pre- and post- ablation was left at the discretion of the clinician. Arrhythmia recurrence was defined as AF, atrial flutter (AFL) or atrial tachycardia (AT) lasting > 30 s on 12-lead ECG, ambulatory monitoring, or implantable device, as recommended by contemporary guidelines [7]. Patients who were continued on AAD after the 3-month blanking period were censored from the analysis assessing recurrence of atrial arrhythmias off AAD.

Adverse events were recorded in the registry and included access site complications (i.e. bleeding, groin hematoma, pseudoaneurysm and arteriovenous fistula), cardiac perforation or tamponade, stroke or transient ischemic attack, pericarditis, myocardial infarction, atrioesophageal fistula, phrenic nerve paralysis, and pulmonary vein stenosis. As part of the registry, follow-up arrhythmia monitoring was pre-specified and was recommended as a 12-lead ECG at each follow-up visit, along with routine ambulatory ECG monitoring (24-h Holter monitor, extended ambulatory ECG monitoring, or event monitoring) in all patients at 6 months, 1 year, and 2 years after ablation and additional ambulatory ECG monitoring to evaluate for arrhythmia recurrence in the presence of suggestive symptoms, which was consistent with consensus guidelines and updated consensus guidelines at the time of the registry [7, 8].

### Radiofrequency ablation procedure

Informed consent was obtained prior to all ablation procedures. General anesthesia was used in all cases. Intravenous heparin was used to target an activated clotting time of 300–400 s. A transseptal puncture was performed under direct visualization with intracardiac echocardiography. Pulmonary vein isolation was performed using segmental, circumferential, or both types of ablations at the discretion of the operator. Closed and open irrigated and non-contact and contact force sensing catheters were used at the discretion of the operator. Electroanatomic mapping systems were used in all cases (CARTO™, Biosense-Webster Inc, Diamond Bar, CA, or Ensite™, St Jude Medical, Inc, Minneapolis, MN). Pulmonary vein entrance and exit block were confirmed with use of a circular catheter, after which adenosine and isoproterenol were administered at the operator’s discretion. Isoproterenol infusion was given at a rate of 20 mcg/min between 3 and 20 min or at the discretion of the operator, and additional triggers and targets were ablated based on criteria established in previous studies, but were ultimately at the discretion of the operator [9, 10]. Triggers were defined as ectopic foci that induced atrial fibrillation whereas targets were areas of reconnection or ectopic foci that did not induce atrial fibrillation. Additional lesion sets including cavotricuspid isthmus line, left atrial roof line, mitral isthmus line, coronary sinus ablation, and ablation of complex fractional atrial electrograms were performed at the discretion of the operator.

### Statistical analysis

Continuous variables are presented by group as the mean ± one standard deviation for normally distributed variables and the median with 25th and 75th percentiles for variables that were not normally distributed. Comparison between all groups was done using the non-parametric Kruskal–Wallis tests. Comparisons between groups were performed using the Student *t* test if the data were normally distributed or the Wilcoxon rank sum test was used if the data were not normally distributed. Categorical variables were reported as count and percentage, with the χ^2^ or Fisher exact test (expected cell counts < 5) used for comparisons.

Recurrence of atrial arrhythmias at final follow up was analyzed using the Kaplan–Meier method with a 3-month blanking period and log-rank significance testing. Unadjusted and adjusted Cox proportional hazards modeling was used to analyze recurrence of atrial arrhythmias with a 3-month blanking period, results are presented as hazard ratios (HRs) with 95% confidence intervals (CIs). Patients who were lost to follow-up were censored at the date of last known follow-up. All of the covariates except the echocardiographic parameters listed in Table 1 were included in the adjusted model, which were selected based on a clinically plausible association of the categorical predictor variable (isoproterenol infusion) with recurrence of the primary outcome of recurrent atrial arrhythmias. Missing values were minimal and roughly equivalent between groups for all variables and were thus omitted. Analyses were performed using Stata 11 (StataCorp, LLC, College Station, TX) statistical software. A *p* < 0.05 was considered statistically significant.

**Table 1:** Baseline characteristics

	Isoproterenol (*n* = 235)	No isoproterenol (*n* = 79)	*P* value
Follow-up duration (months)	43.6 (23.3, 57.6)	36.7 (8.9, 59.2)	0.073
Age (years)	65.1 (58.6, 71.7)	68.4 (58.1, 73.1)	0.146
Male	82 (34.9)	26 (32.9)	0.748
BMI (kg/m^2^)	28.0 (25.0, 32.1)	27.8 (24.7, 30.9)	0.457
**AF type**			< 0.001
Paroxysmal	169 (73.5)	31 (41.9)	
Persistent	61 (26.5)	43 (58.1)	
CHA_2_DS_2_VASc	2.0 (1.0, 3.0)	2.0 (1.0, 4.0)	0.276
**Comorbidities**			
HF	40 (17.1)	15 (19.2)	0.407
HTN	137 (58.5)	51 (65.4)	0.285
HLD	96 (41.0)	33 (42.3)	0.842
DM	24 (10.3)	9 (11.5)	0.750
COPD	9 (3.8)	4 (5.1)	0.624
OSA	28 (12.0)	11 (14.1)	0.621
Prior CVA	18 (7.7)	11 (14.1)	0.091
CAD	37 (15.8)	16 (20.5)	0.338
ESRD	1 (0.4)	1 (1.3)	0.417
Smoker	66 (28.2)	26 (33.3)	0.390
**Echocardiographic parameters**			
LVEF (%)	63 (57, 67)	60 (55, 66)	0.186
LAD (cm)	4.11 ± 0.56	4.27 ± 0.72	0.110
LVEDD (cm)	4.81 ± 0.57	4.88 ± 0.69	0.493
MVR	88 (50.3)	20 (45.5)	0.567
**Cardiovascular medications**			
Beta-blocker	141 (60.0)	41 (52.6)	0.249
Calcium channel blocker	65 (27.7)	23 (29.5)	0.756
ACE-I	39 (16.6)	20 (25.3)	0.086
ARB	36 (15.3)	17 (21.5)	0.203
Aldosterone antagonist	4 (1.7)	8 (10.1)	0.001
Digoxin	23 (9.8)	4 (5.1)	0.204
Aspirin	89 (37.9)	22 (28.2)	0.122
Theinopyridine	7 (3.0)	1 (1.3)	0.411
Coumadin	58 (24.7)	26 (33.3)	0.135
Apixaban	24 (10.2)	4 (5.1)	0.173
Dabigatran	27 (11.5)	10 (12.8)	0.752
Rivaroxaban	65 (27.7)	18 (23.1)	0.427
**AAD preablation**			
None	84 (35.7)	23 (29.5)	0.383
Flecainide	44 (18.7)	19 (24.4)	0.282
Propafenone	15 (6.4)	5 (6.4)	0.993
Sotalol	49 (20.9)	18 (23.1)	0.678
Dronedarone	8 (3.4)	5 (6.4)	0.249
Amiodarone	29 (12.3)	6 (7.7)	0.259
Dofetillide	6 (2.5)	2 (2.6)	0.996
**Device preablation**			
PPM	11 (4.7)	6 (7.7)	0.313
ICD or CRT-D	6 (2.6)	3 (3.8)	0.558

Values are presented as median (Q1, Q3) for continuous variables or *n* (%) for categorical variables

*ACE* angiotensin receptor blocker, *BMI* body mass index, *CHA2DS2VASc* risk score for thromboembolic events, *COPD* chronic obstructive pulmonary disease, *CRT-D* cardiac resynchronization therapy defibrillator, *CVA* cerebrovascular accident, *HF* heart failure, *ICD* implantable cardiac defibrillator, *LAD* left atrial diameter, *LVEDD* left ventricular end diastolic diameter, *LVEF* left ventricular ejection fraction, *PPM* permanent pacemaker

## Results

### Patient characteristics

A total of 314 patients underwent de novo radiofrequency catheter ablation during the study period with a median follow-up of 43.6 (23.3,57.6) months in the patients who received isoproterenol during ablation and 36.7 (8.9, 59.2) in the patients who did not (*p* = 0.073). Baseline characteristics are summarized in Table 1. Of the analyzed cohort, 74.8% (*n* = 235) received isoproterenol during ablation and 25.2% (*n* = 79) did not. Patient who received isoproterenol were more likely to have paroxysmal atrial fibrillation (73.5% vs 41.9%; *p* < 0.001) and less likely be prescribed an aldosterone antagonist (1.7% vs 10.1%; *p* = 0.001).

Ablation characteristics are summarized in Table 2. The isoproterenol group had shorter fluoroscopy times [65 min (53, 81) vs 72 min (57, 99); *p* = 0.041]. The types of additional ablations performed between groups were similar, with the exception that less left atrial roof lines were drawn in the isoproterenol group (22.1% vs 34.6%; *p* = 0.028).

**Table 2:** Comparison of ablation characteristics and complications

	Isoproterenol (*n* = 235)	No isoproterenol (*n* = 79)	*P* value
Total procedure time (minutes)	250 (207, 296)	259 (211, 295)	0.432
Total fluoroscopy time (minutes)	65 (53, 81)	72 (57, 99)	0.041
**Additional ablation**			
Mitral isthmus line	18 (7.7)	10 (12.8)	0.166
LA roof line	52 (22.1)	27 (34.6)	0.028
CFAE ablation	9 (3.8)	3 (3.8)	0.995
CTI ablation	223 (94.9)	74 (93.7)	0.678
**Procedural complications**			
Access site complication‡			
Access site bleeding	17 (7.2)	7 (8.9)	0.638
Groin hematoma	9 (3.8)	2 (2.5)	0.587
Groin pseudoaneurysm	1 (0.4)	0 (0.0)	0.561
Groin arteriovenous fistula	1 (0.4)	0 (0.0)	0.561
Cardiac perforation/tamponade	0 (0.0)	2 (2.5)	0.014
Stroke/TIA	18 (7.7)	11 (14.1)	0.091
Pericarditis	0 (0.0)	1 (1.3)	0.084
Other complications^§^			
Myocardial infarction	0 (0.0)	0 (0.0)	NA
Atrioesophageal fistula	0 (0.0)	0 (0.0)	NA
Phrenic nerve paralysis	0 (0.0)	0 (0.0)	NA
Pulmonary vein stenosis	0 (0.0)	0 (0.0)	NA

Values are presented as median (Q1, Q3) for continuous variables or *n* (%) for categorical variables

^‡^Access site complications included access site bleeding, groin hematoma, groin pseudoaneurysm, and groin arteriovenous fistula

^§^Other complications included myocardial infarction, atrioesophageal fistula, phrenic nerve paralysis, and pulmonary vein stenosis

Among the patients that received isoproterenol, 11 (4.7%) had additional triggers/targets identified (Table 3). Eight of these patients received adenosine prior to isoproterenol infusion, two of which had reconnection identified afterwards. Among these, 1 was not intervened on, 1 underwent direct current cardioversion for triggered atrial fibrillation, and 9 had additional ablations performed of which 7 were successful in terminating the ectopy or atrial fibrillation induced by isoproterenol infusion. Additional ablation that resulted in successful termination of atrial fibrillation or elimination of triggering ectopy included ablation near the left upper pulmonary vein in one patient (just proximal to previous circumferential pulmonary vein ablation lesions), ablation of both left upper and lower pulmonary veins in two patients due to reconnections, ablation of the left lower and right upper pulmonary veins in one patient due to reconnections, ablation of the right lower pulmonary vein after reconnection in one patient, ablation of ectopy from the right pulmonary vein carina in one patient, and ablation of ectopy in the right atrium in one patient (see Table 3). One patient with unsuccessful termination underwent additional ablation of the lower right septum and adjacent to the right upper pulmonary vein, followed by further ablation of the right atrium that had to be stopped due to proximity to the sinus node and phrenic nerve. The other patient with unsuccessful termination had further ablation near the posterior antrum of the left upper pulmonary vein that had to be stopped prior to complete ablation due to esophageal temperature rise.

**Table 3:** Characteristics of patients with triggers or targets identified after isoproterenol infusion

Patient	Trigger or target	Arrhythmia induced	Location	Additional comments
1	Trigger	Premature atrial contractions triggering atrial fibrillation	Antral left upper pulmonary vein	
2	Trigger	Premature atrial contractions	Lower right septum, right upper pulmonary vein and right atrium	Abandoned due to proximity to sinus node and phrenic nerve
3	Target	Reconnection	Left pulmonary veins	
4	Trigger	Premature atrial contractions triggering atrial fibrillation	Posterior antrum of left upper pulmonary vein	Unable to completely ablate due to esophageal temperature rises
5	Target	Reconnection	Right lower pulmonary vein	Reconnection of pulmonary vein
6	Target	Atrial fibrillation	Left lower and right upper pulmonary veins	Reconnection of pulmonary veins
7	N/A	Atrial fibrillation	N/A	Terminated via direct current cardioversion
8	Trigger	Premature atrial contractions	Right atrium	Atrial ectopy, no further ablation pursued
9	Trigger	Premature atrial contractions	Right pulmonary vein carina	
10	Trigger	Supraventricular tachycardia	Right atrium	Near sinus node
11	Target	Reconnection	Left pulmonary veins	Reconnection

### Ablation outcomes

**Fig. 1:**
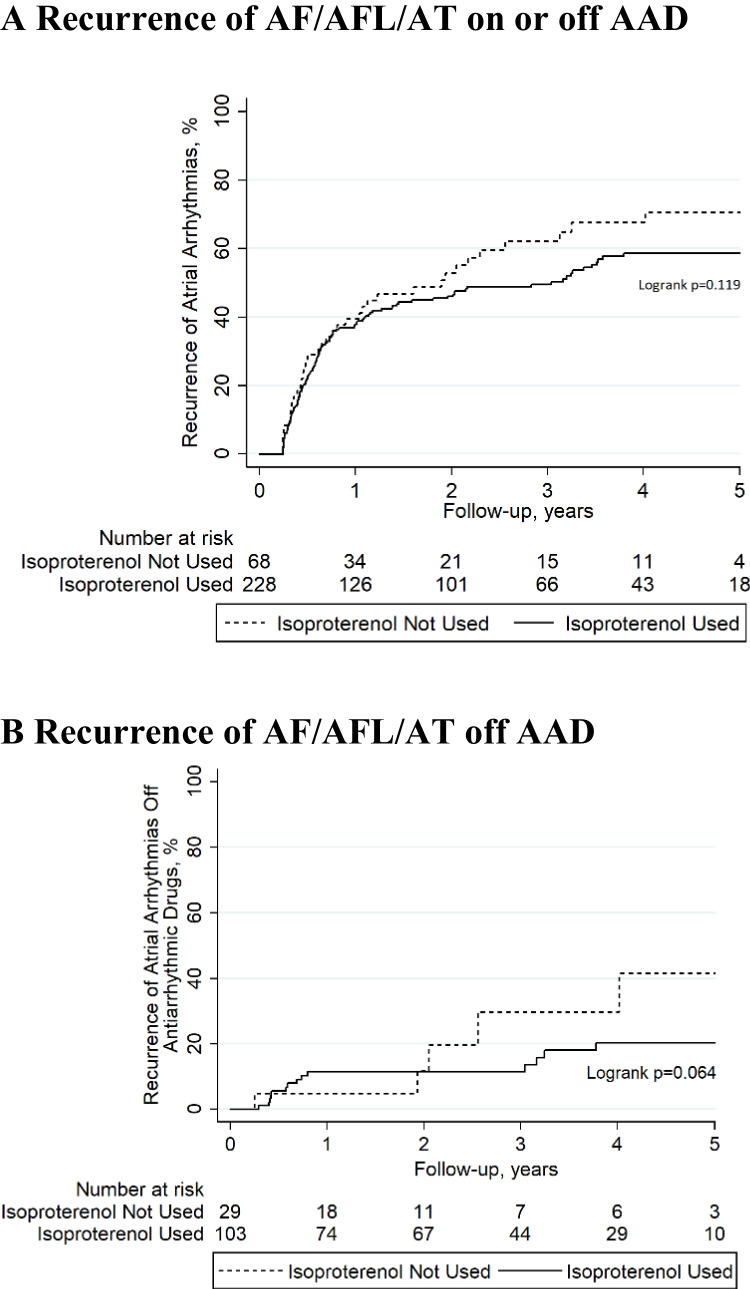
Kaplan–Meier plots of **A** long-term recurrence of atrial arrhythmias on or off antiarrhythmic drugs (excluding a 3-month post-procedural blanking period), and **B** long-term recurrence of atrial arrhythmias off antiarrhythmic drugs (excluding a 3-month post-procedural blanking period). Patients who received isoproterenol during the index ablation procedure and those that did not are compared. Abbreviations: AAD = antiarrhythmic drug; AF = atrial fibrillation; AFL = atrial flutter; AT = atrial tachycardia

There was less incidence of cardiac perforation or tamponade in the isoproterenol group relative to those who did not receive isoproterenol (0.0% vs 2.5%; *p* = 0.014), but event rates were overall very low (Table 2). Recurrence of atrial arrhythmias on or off AAD (49.4% versus 51.9%; log rank *p* = 0.119) and off AAD (51.4% versus 59.7%; log rank *p* = 0.064) was statistically similar in the isoproterenol group compared to the no isoproterenol group during follow-up (Fig. 1 A and B). Patients were on AAD after the 3-month blanking period in 32.3% (*n* = 76) of patients who received isoproterenol and in 38.0% (*n* = 30) of patients who did not receive isoproterenol (*p* = 0.360). There was no statistically significant difference in patients who underwent repeat ablations in the isoproterenol group relative to the no isoproterenol group (0.9% versus 0.0%; *p* = 0.414).

**Fig. 2:**
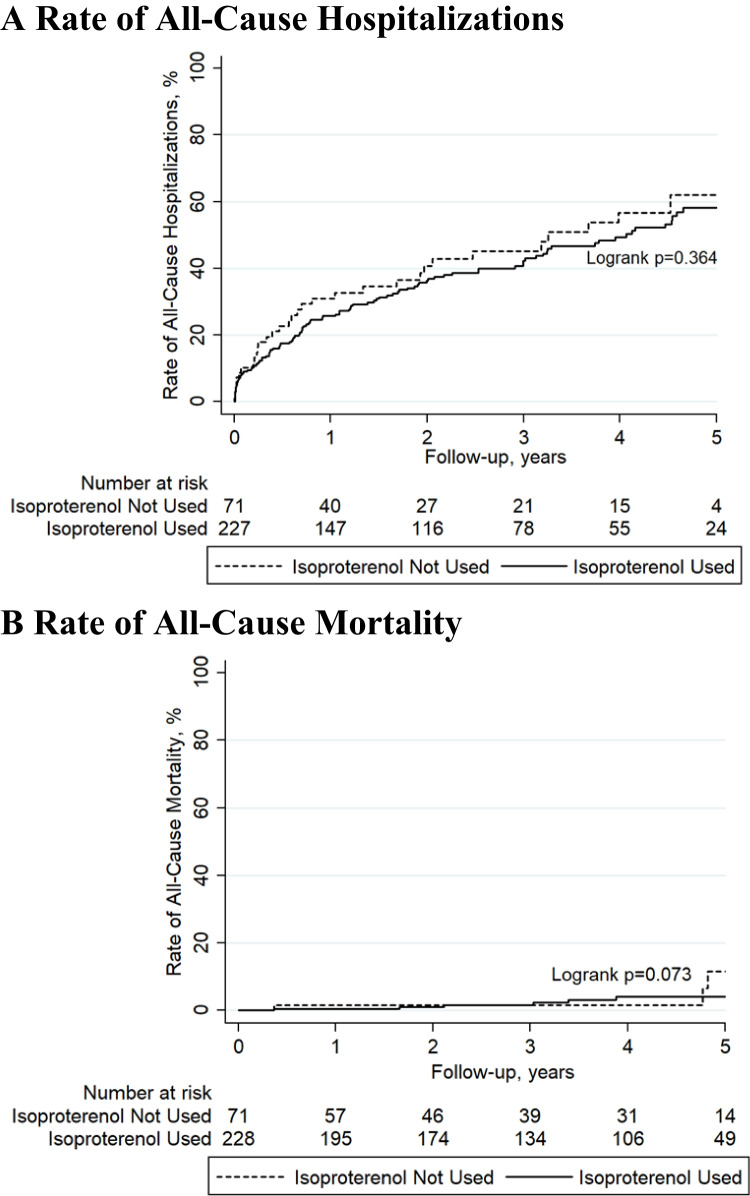
Kaplan–Meier plots of **A** long-term rate of all-cause hospitalizations and **B** long-term rate of all-cause mortality. Patients who received isoproterenol during ablation and those that did not are compared. Abbreviations: AAD = antiarrhythmic drug; AF = atrial fibrillation; AFL = atrial flutter; AT = atrial tachycardia

Rates of all-cause hospitalizations (44.7% versus 42.3%; log rank *p* = 0.364) and all-cause mortality (2.6% versus 6.4%; log rank *p* = 0.073) were also statistically similar in the isoproterenol group relative to the no isoproterenol group over all follow-up (Fig. 2 A and B).

Hazard ratios with multivariable adjustment for potential confounders and respective confidence intervals for recurrence of atrial arrhythmias and all-cause hospitalizations and mortality are summarized in Table 4 and showed no association of isoproterenol infusion with these clinical outcomes, after adjustment.

**Table 4:** Adjusted hazard ratios and confidence intervals

Adjusted HR (isoproterenol vs no isoproterenol)	*P* value
Recurrence of AF/AFL/AT on or off AAD	
0.92 (95% CI 0.58–1.46)	0.714
Recurrence of AF/AFL/AT off AAD	
0.24 (95% CI 0.05–1.18)	0.078
Rates of all-cause hospitalizations	
1.00 (0.60–1.67)	0.986
Rates of all-cause mortality	
0.14 (0.01–3.52)	0.229

*AF* atrial fibrillation, *AFL* atrial flutter, *AT* atrial tachycardia, *CI* confidence interval, *HR* hazard ratio

A subgroup analysis that only included patients with paroxysmal atrial fibrillation also showed no significant difference in recurrence of atrial arrhythmias on or off AAD [adjusted hazard ratio (AHR) 0.68 (95% CI 0.34–1.37); *p* = 0.276], recurrence of atrial arrhythmias off AAD [AHR 0.64 (95% CI 0.33–1.26); *p* = 0.195], all-cause hospitalizations [AHR 0.85 (95% CI 0.42–1.74); *p* = 0.662], or all-cause mortality [AHR 0.24 (95% CI 0.00–54.04); *p* = 0.609].

## Discussion

In this observational registry cohort study, use of isoproterenol was safe, but only identified triggers and targets in a small minority of cases and was not associated with a reduction in recurrence of atrial arrhythmias. However, this result may be confounded by the significantly higher proportion of patients with paroxysmal atrial fibrillation in the isoproterenol group. Still, this would be expected to bias the results in favor of the isoproterenol group and there was not a reduction in recurrence of atrial arrhythmias with isoproterenol use despite this. However, the higher proportion of persistent atrial fibrillation in the patients who did not receive isoproterenol could explain the higher percentage of non-pulmonary vein ablation and the lack of a significant difference in procedure times between groups. Still, there was no difference even after a sub-group analysis of patients with paroxysmal atrial fibrillation or after results were adjusted for multiple covariates, including the type of atrial fibrillation.

The mechanism by which isoproterenol induces triggers for atrial fibrillation is likely multifactorial. Isoproterenol is a synthetic amine with β_1_-agonist and β_2_-agonist activity, the latter of which results in vasodilation and is responsible for the potential hypotension side effect, often necessitating the use of an α1 receptor agonist, such as phenylephrine, to augment blood pressure. Isoproterenol decreases sinus cycle length, shortens the refractory period, releases calcium from the sarcoplasmic reticulum, and promotes early after-depolarizations, automaticity, and triggered activity [11–14]. Interestingly, AF induced by isoproterenol has been shown to persist beyond the point of complete washout, which suggests that once AF is triggered, self-perpetuating mechanisms, such as pulmonary vein tachycardias, were activated [15].

The most common triggers identified after high dose isoproterenol infusion were adjacent to the pulmonary veins. Other sites of triggers were successfully ablated in the majority of cases, but occasionally were limited by proximity to vital areas, such as the sinus node, or due to esophageal temperature rises. Notably, there was a lack of triggers identified in other areas such as the coronary sinus or left atrial appendage.

The results of previous studies are mixed. Crawford et al. prospectively used isoproterenol to induce AF prior to ablation and then again after ablation to identify residual triggers. They found that AF was inducible pre-ablation with isoproterenol in 87% (*n* = 97) of patients at a mean dose of 15 ± 5 μg/min (with the remainder being induced via right atrial pacing). AF was reinduced post-ablation by isoproterenol in 18% (*n* = 15), where AF was terminated with additional ablation at the pulmonary vein ostia in 6% (*n* = 5) of patients and left atrial roof in 2% (*n* = 2). The other 10% (*n* = 8) had to be cardioverted to restore sinus rhythm after further ablation was unsuccessful in terminating AF. Among these 8 patients, additional ablation was performed along the posterior mitral annulus and inferior wall in 5 patients, along the rim between the left-sided pulmonary veins and left atrial appendage in 4 patients, pulmonary vein ostia in 2 patients, base of left atrial appendage in 2 patients, and anterior left atrium in 2 patients [16]. Elayi et al. prospectively looked at non-pulmonary vein triggers after administering adenosine followed by isoproterenol. They found non-pulmonary vein triggers in 17% (*n* = 32) of patients, all of which were ablated successfully. The three most common sites ablated were the coronary sinus, septum and left atrial appendage. Isoproterenol revealed 86% of the non-pulmonary vein triggers. Of note, pulmonary vein reconnection was seen in 4.9% (*n* = 19) of patients after drug challenge, of which 1.6% (*n* = 6) had reconnections with pulmonary vein triggers inducing AF [17]. Sakamoto et al. also prospectively studied the efficacy of isoproterenol after administration of adenosine. However, isoproterenol was only infused in cases where adenosine did not reveal reconnection of the pulmonary veins or if the reconnection was transient. In 13% (*n* = 13) of patients, pulmonary vein reconnection was seen after isoproterenol infusion requiring additional ablation. Non-pulmonary vein triggers were identified in 23% (*n* = 23) patients after administration of isoproterenol. Among these, 5 were at the superior vena cava, 5 at the right atrial septum, 3 in the right atrium, 2 in the left atrial septum, 2 in the anterior left atrium, 2 in the posterior left atrium, 2 in the coronary sinus, one at the tricuspid valve, and one at the crista terminalis [18].

Overall, the prevalence of isoproterenol induced triggers/targets in our study was found to be lower than some cohorts and equal to others. While isoproterenol has been used to assess for residual AF triggers in multiple studies and has been shown to induce AF in a dose-dependent manner [5], it has not been definitively shown to be associated with differential outcomes after ablation of AF. Use of isoproterenol was not associated with a reduction in recurrence of atrial arrhythmias in this study.

These findings are significant as isoproterenol can result in hypotension and is expensive, especially during AF ablation where high doses are required often necessitating two vials of the drug. Through several pharmaceutical acquisitions, the price of isoproterenol per milligram has increased from $26 to $1790 from 2012 to 2015, with the price continuing to steadily rise [6]. This means that use of isoproterenol could cost thousands of dollars per ablation procedure without demonstrably reducing recurrence of atrial arrhythmias according to this study. This should prompt consideration of alternative agents to elicit atrial triggers during ablation of AF. Recently, high-dose dobutamine has been studied as a promising alternative, as it results in less hypotension and is significantly cheaper [19].

### Study limitations

There are some limitations to interpreting the data presented in this study. First, the generalizability may be limited given that this study involved a single-center and is a retrospective study. Second, it could be argued that patients selected to receive isoproterenol had more complicated ablation procedures, but results were adjusted for baseline covariates and most ablation characteristics were not statistically significant between groups. Third, there was no standardized protocol for duration of isoproterenol infusion as it was left to the discretion of the operator. Fourth, some patients were lost to follow-up after 2–3 years, which may falsely inflate recurrence rates.

## Conclusion

In this observational registry study, use of isoproterenol was safe, but was not associated with a reduction in recurrence of atrial arrhythmias. In those infused with isoproterenol, a minority (4.7%) actually developed an additional target or trigger that was ablated. There were also no significant differences in rates of all-cause hospitalizations and mortality regardless of isoproterenol use.

## Abbreviations

AAD: Antiarrhythmic drug
AF: Atrial fibrillation
AFL: Atrial flutter
AT: Atrial tachycardia
CI: Confidence interval
HR: Hazard ratio
